# Clinical and neuroimaging disparity between Chinese and German patients with cerebral small vessel disease: a comparative study

**DOI:** 10.1038/s41598-019-55899-w

**Published:** 2019-12-27

**Authors:** Junlong Shu, Hermann Neugebauer, Fan Li, Dorothée Lulé, Hans-Peter Müller, Jing Zhang, Albert C. Ludolph, Yining Huang, Jan Kassubek, Wei Zhang

**Affiliations:** 1 Department of Neurology, Peking University First Hospital, Xishiku Street 7, Beijing, 100034 China; 2 Department of Neurology, Ulm University Clinic, Oberer Eselsberg 45, Ulm, 89081 Germany; 3 Present Address: Department of Neurology, University Hospital Würzburg, Josef-Schneider-Str. 11, 97080 Würzburg, Germany

**Keywords:** White matter disease, Stroke, Dementia

## Abstract

Ethnic disparity of cerebral small vessel disease (CSVD) has been reported previously but understanding of its clinical-anatomical is sparse. Two cohorts of CSVD patients from Peking University First Hospital, China and University Hospital of Ulm, Germany were retrospectively collected between 2013 and 2017. Visual rating scales and semiautomatic computer-assisted quantitative analysis were used to describe the neuroimaging features of CSVD, including lacunes, enlarged perivascular spaces, white matter changes and microbleeds. After exclusion of confounding neurological disorders, 165 out of 220 Chinese and 86 out of 98 German patients’ data were analyzed. Mean age of patients was 64.0 ± 11.9 years in China and 73.9 ± 10.3 years in Germany. Cognitive deficits were more prominent in the German group, mainly in the cognitive domains of language and delayed recall. Neuroimaging comparison showed that lacunes were more common and white matter lesion load was more severe in German than Chinese patients. Spatial distribution analysis suggested that Chinese patients showed more deep and infratentorial lesions (microbleeds and lacunes), while lesions in German patients were more frequently located in the lobes or subcortical white matter. In conclusion, different age of onset and anatomical distribution of lesions exist between Chinese and German CSVD patients in the observed population.

## Introduction

Cerebral small vessel disease (CSVD) is a syndrome defined by clinical presentation and neuroimaging with a heterogeneous etiology. The age and hypertension-related small vessel disease is the most common form^1^. From a clinical point of view, CSVD is a major cause of stroke and vascular cognitive impairment. In addition, progressive dysfunction of gait, swallowing and sphincters lead to disability and loss of autonomy^1,2^, and the socioeconomic impact is tremendous^3^. As the small vessels can currently not be visualized *in vivo*, the typical parenchymal lesions in neuroimaging are thought to be directly related to impairment of small cerebral vessels. As markers of small vessel disease, recent small subcortical infarct, lacune of presumed vascular origin, white matter hyperintensity of presumed vascular origin, enlarged perivascular spaces, cerebral microbleed and brain trophy^4^ have been adopted^1,3^. The pathogenesis of these changes is not well understood, but is thought to be diffuse cerebrovascular endothelial failure as a result of a combination of traditional vascular risk factors, socioeconomic risk factors, genetic susceptibility and as yet undetermined environmental factors^1,3^.

Ethnic disparity of CSVD has been reported previously^5–10^. A hospital-based study from the Netherlands showed that white stroke patients had higher odds for carotid artery stenosis while Black and Asian stroke patients more often presented with lacunar infarcts^10^. In the South London Ethnicity and Stroke Study, small vessel disease associated stroke and white matter hyperintensities were markedly increased in Black Africans versus White stroke patients, while Black Caribbean appeared to have an intermediate risk^5^. In a multiethnic stroke population from Florida, a higher proportion of lacunar stroke was found in Caribbean Blacks, African Americans, and Caribbean Hispanics, compared with non-Hispanic Whites^6^. The population-based Auckland Regional Community Stroke Study found Maori/Pacific and Asian/other people at higher risk of primary intracerebral hemorrhage compared to New Zealand/Europeans^9^. In addition, emerging evidence exists that - compared with white populations of European origin, Chinese populations have a higher proportion of stroke attributable to intracerebral hemorrhage and lacunar ischemic stroke^7,8^. All these studies yield similar results, showing that Black and Yellow patients share a higher susceptibility to CSVD than White patients^5,7–9^. These differences are commonly explained by a different prevalence of vascular risk factors^9^, yet undetermined genetic or environmental risk factors may also account for it. However, these previous studies had their focus on the prevalence of CSVD and the distribution of risk factors, while only few studies^11,12^ reported on differences in clinical phenotype and anatomical distribution of CSVD on brain imaging between different ethnic groups. From a pathophysiological point of view, the clinical-anatomical correlation in particular might provide a deeper insight into the mechanisms of CSVD.

Owing the support from the joint project of Peking University Health Science Center (PUHSC) and University of Ulm, we were able to acquire patients’ data from both countries and to conduct a retrospective study to explore the clinical and neuroimaging disparities of CSVD between Chinese and German patients.

## Methods

### Study population

Two cohorts of CSVD patients from Peking University First Hospital and University Hospital of Ulm (University and Rehabilitation Clinics Ulm, RKU), were recruited for the study. Inclusion criteria for both groups were recent lacunar stoke, transient ischemic attack (TIA), intracerebral hemorrhage (ICH) or other symptoms such as progressive gait disorder and cognitive decline due to vascular factors, with one or more characteristic imaging changes of CSVD in magnetic resonance imaging (MRI) according to current standards, including recent small subcortical infarct, lacune of presumed vascular origin, white matter hyperintensity of presumed vascular origin, enlarged perivascular space or cerebral microbleed^4^. The Chinese patients were recruited between 2013 and 2017 from the Dept. of Neurology wards and outpatient clinic of the Peking University First Hospital. German patients were recruited between 2015 and 2017 from the stroke unit and outpatient clinic of the university hospital of Ulm. Informed consents were obtained from all participants. This research was approved by the Ethics Committee of Peking University (IRB00001052-17018) and Ulm University (#295/17), and performed in accordance with the declaration of Helsinki. Patients with large vessel stenosis, which was defined as a stenosis more than 50% detected by carotid ultrasound/transcranial doppler sonography (TCD) or magnetic resonance angiography (MRA) in any large or medium artery supplying brain tissue, or suspicion of cerebral amyloid angiopathy (CAA) or Alzheimer disease (AD) from clinical course or imaging changes were excluded from the final analysis.

### Clinical data collection

Demographic information of the patients such as age, sex, education, and body mass index (BMI) were collected. Common vascular risk factors including hypertension, diabetes mellitus, dyslipidemia, coronary heart disease, atrial fibrillation, chronic kidney disease, level of serum homocysteine (China: HLPC, Germany: Enzyme-Cycling-Assay), smoking, and family stroke history were documented on the first visit of the patient. Color Doppler carotid ultrasound, TCD and MRA were used to reveal the extra- and intracranial artery changes. 143 of the patients accepted neuropsychological assessment using the Mini-mental State Examination (MMSE). In a subgroup of patients (N = 96), the Montreal Cognitive Assessment (MoCA) was conducted additionally. These scales were assessed during the follow-up at least 2 weeks after stroke, in order to rule out the effect of acute stroke on cognitive function.

### MR scanning and measuring

Each patient had undergone head MR scan, with sequences including T1-weighted, T2-weighted, fluid attenuated inversion recovery (FLAIR), diffusion weighted imaging (DWI) and T2^*^-weighted gradient-recalled echo (GRE). The MRI data of Beijing were acquired on two MR scanners (General Electric Medical Systems 1.5 T, Philips Medical Systems 3.0 T), with axial slices and slice thickness of 6.0 mm. The MRI data of Ulm were acquired on a 1.5 T MR scanner (Symphony, Siemens Medical), with coronal slices for FLAIR image, axial slices for others, and thickness of 5.0 mm.

Two independent specifically trained raters analyzed the MRI data. The intraclass correlation coefficient (ICC) of intrarater reliability was 0.98 [95% CI 0.95–1.00] and interrater reliability was 0.92 [95% CI 0.86–0.95]. Key neuroimaging markers including lacunes, enlarged perivascular spaces, white matter changes and microbleeds were evaluated, followed the definition of STandards for ReportIng Vascular changes on nEuroimaging (STRIVE)^4^. The presence, number and location of the lacunes were recorded. For lacune counting, different areas were documented in the right and left hemispheres separately, i.e., frontal, parietooccipital, temporal, corpus callosum, internal capsule, external capsule, basal ganglia, thalamus, brainstem and cerebellum. Enlarged perivascular spaces were assessed on T2-weighted image at the basal ganglia and corona radiate level. According to severity, we classified it into three types: A. none, B. visible (round/ovoid, <3 mm cavity at basal ganglia level or few linear changes at corona radiate level), and C. diffuse (honeycomb-like change at basal ganglia level or cloth-like change at corona radiate level). White matter changes (WMC) were assessed qualitatively and quantitatively. The age-related white matter changes (ARWMC) scale^13^ was adopted to rate the severity and distribution of the lesions. A semiautomatic analysis software^14^ was applied to extract and calculate the volume of white matter change. For microbleeds, we used the Microbleed Anatomical Rating Scale (MARS)^15^ to record information of its presence, number and distribution.

### Statistical analysis

Statistical analyses were performed using the Statistical Package for Social Sciences version 24.0 (IBM SPSS Statistics, Armonk, N.Y., USA). The Shapiro-Wilk test was used to examine the distribution of data. Quantitative data are expressed as means and standard deviations (SD) or median depending on the distribution of the values. Categorical data are described as frequencies and ratios. The t-test was used for the comparison of age, and Mann-Whitney U test was used for other non-normal distribution data. We used Pearson’s chi-squared test to analyze the difference of categorical data. Regression analysis was conducted for evaluating whether there is a significant difference after correcting the effects of confounding factors such as age. P < 0.05 was considered significant.

## Results

### Demographic and risk factors

Out of a total of 318 CSVD patients, 220 patients were Chinese and 98 cases were German. Fifty-five patients from China and 3 patients from Germany were excluded for large vessel stenosis, and 9 patients from Germany were excluded for suspicion of CAA or AD. The data of 165 Chinese patients and 86 German patients were included in the final analysis. In the Chinese group, 121(73%) patients manifested with lacunar stroke or TIA, 35(21%) with ICH, and 9(6%) presented with other symptoms of CSVD. In the German group, there were 58(67%) lacunar strokes or TIAs, 11(13%) ICHs, and 17(20%) presented with other symptoms of CSVD. The average age of Chinese patients was 64.0 ± 11.9 years, while the German patients were about 10 years older at onset (73.9 ± 10.3 years), with a similar male proportion (68.5% versus 59.3%, P = 0.15).

With respect to risk factors, more Chinese patients were diagnosed with hyperlipidemia (54.8% vs. 35.9%, P = 0.007) and smoking (42.5% vs. 7.3%, P < 0.01). Defined as serum homocysteine levels above 15 umol/L, German group had a significantly higher proportion of hyperhomocysteinemia (79.7%), in contrast to the Chinese group (31.5%). In addition, the proportion of obesity (BMI ≥ 30 kg/m2) was 20.5% in German group, while the proportion in China group was only 9.8%, which also presented statistical difference (P = 0.03). The baseline characteristics and risk factors of the both groups are shown in Table 1.

**Table 1:** Baseline characteristics and risk factors of the two cohorts.

		Country		P
		Chinese (N = 165)	German (N = 86)	
Clinic course	Lacunar stroke or TIA	121 (73%)	58 (67%)	
	ICH	35 (21%)	11 (13%)	
	Other	9 (6%)	17 (20%)	
Sex (Male)		113 (68.5%)	51 (59.3%)	0.15
Age (Mean ± SD)		64.0 ± 11.9	73.9 ± 10.3	<0.01
Hypertension		146 (88.5%)	82 (95.3%)	0.07
Diabetes		45 (30.8%)	18 (20.9%)	0.10
Hyperlipidemia		80 (54.8%)	28 (35.9%)	0.01
Coronary Heart Disease		23 (15.8%)	14 (16.3%)	0.92
Atrial Fibrillation		20 (13.7%)	13 (15.1%)	0.77
Hyperhomocysteinemia		46 (31.5%)	63 (79.7%)	<0.01
Smoking		62 (42.5%)	6 (7.3%)	<0.01
Obesity		13 (9.8%)	17 (20.5%)	0.03
Chronic Kidney Disease		11 (7.5%)	13 (15.5%)	0.06
Family history of stroke		41 (28.1%)	20 (23.5%)	0.45

Abbreviations: TIA = Transit Ischemic attack, ICH = Intracranial heamorrhage, SD = Standard Deviation.

### Cognitive function

Sixty Chinese and 83 German patients were assessed by use of the MMSE scale, and among them, 59 Chinese and 37 German patients were also assessed with MoCA scale on the same day. The comparison showed that German patients had lower scores than Chinese patients both in MMSE [27(23, 28) vs. 28(26, 29), P < 0.01] and MoCA [21(16, 25) vs. 25(22, 27), P < 0.01]. However, these differences were not significant after adjusting for the confounding effect of age. Cognitive subdomain analysis of MoCA showed that German patients performed worse in language, delayed recall, and spatial orientation (Table 2).

**Table 2:** Comparison of cognitive function in subgroups of Chinese and German patients.

		Chinese	German	P	P^ª^
		(MMSE: N = 60)	(MMSE: N = 83)		
		(MOCA: N = 59)	(MOCA: N = 37)		
MMSE, M (P25, P75)		28 (26, 29)	27 (23, 28)	<0.01	0.26
MOCA^*^, M (P27, P75)		25 (22, 27)	21 (16, 25)	<0.01	0.06
Subdomains	Visuospatial/Executive	4 (3, 5)	4 (2, 5)	0.64	0.48
	Naming	3 (3, 3)	3 (3, 3)	0.86	0.77
	Attention	5 (5, 6)	6 (4, 6)	0.76	0.58
	Language	3 (2, 3)	1 (0, 2)	<0.01	<0.01
	Abstraction	2 (1, 2)	2 (1, 2)	0.47	0.27
	Delayed Recall	2 (1, 4)	0 (0, 1)	<0.01	<0.01
	Orientation	6 (6, 6)	6 (5, 6)	<0.01	<0.01

Abbreviations: M = Median; P25, P75 indicate for 25 and 75th percentile.

MMSE = Mini-mental State Examination.

MoCA = Montreal Cognitive Assessment.

*Corrected for education.

ªAdjusted for age.

### Neuroimaging markers and spatial distribution of CSVD

All the patients had MR scanning, but 12 cases were excluded when evaluating the white matter changes and microbleeds due to poor image quality or lack of necessary sequence (Table 3). The German group presented more lacunes than the Chinese group in both occurrence (76.7% vs. 63.6%, P = 0.03) and number [2(1, 5) vs. 1(0, 3), P < 0.01]. There were, furthermore, significant differences in the severity of white matter changes between the two groups. The median volume of WMC in the German group was 28 ml (14.1, 47.2), nearly twice of the median volume in the Chinese group [14.1 ml (5.1, 35.9)]. Significant differences in the ARWMC scores of both groups confirmed this difference [10(6, 14) vs. 6(3, 11), P < 0.01]. These differences were still significant after adjusting for confounding effect of age. There was no difference found in the enlarged perivascular spaces and presence of microbleeds or MARS scores between the two groups.

**Table 3:** Neuroimaging comparison of Chinese and German patients.

		Chinese		German		P	P^ª^
		N		N			
*Lacune*		165		86			
Presence			105 (63.6%)		66 (76.7%)	0.03	0.03
No. M (P25, P75)			1 (0, 3)		2 (1, 5)	<0.01	<0.01
Regions	Frontal region		155 (47.0%)		197 (62.3%)	<0.01	—
	Parietooccipital region						
	Temporal region						
	Corpus callosum						
	Internal Capsule		122 (37.0%)		97 (30.7%)	0.09	—
	External Capsule						
	Basal ganglia						
	Thalamus		53 (16.1%)		22 (7.0%)	<0.01	—
	Brain Stem						
	Cerebellum						
*Enlarged perivascular space*		165		86			
	None		2 (1.2%)		0 (0%)	0.10	—
	Visible		103 (62.4%)		46 (53.5%)		
	Diffuse		60 (36.4%)		40 (46.5%)		
*White matter change*		163		85			
WMC volume(ml), M (P25, P75)			14.1 (5.1, 35.9)		28.0 (14.1, 47.2)	<0.01	0.04
ARWMC Scores, M (P25, P75)			6 (3, 11)		10 (6, 14)	<0.01	0.01
Regions	Subcortical		5 (3, 8.5)		8 (6, 10)	<0.01	<0.01
	Basal ganglia		1 (0, 3)		1 (0, 4)	0.07	0.15
	Infertentorial		0 (0, 0.5)		0 (0, 0)	0.40	0.49
*Microbleed*		157		82			
Presence, N (%)			64 (40.8%)		37 (45.1%)	0.52	0.30
MARS Scores, M (P25, P75)			0 (0, 2)		0 (0, 4)	0.66	0.42
Regions	Lobe		215 (40.0%)		196 (62.4%)	<0.01	—
	Deep		240 (44.6%)		79 (25.2%)	<0.01	—
	Infertentorial		83 (15.4%)		39 (12.4%)	0.23	—

Abbreviations: N = Number; M = Median; P25, P75 indicate for 25 and 75th percentile;

WMC = White matter change; ARWMC = Age-related white matter change;

MARS = Microbleed anatomical rating scale.

ªAdjusted for age.

Taking each lacune and microbleed into account, we analyzed its spatial distribution among different regions, and median ARWMC scores of different regions were calculated. It showed that most of the lacunes located in the subcortical regions (frontal area, parietooccipital area, temporal area, and corpus callosum) (47.0% in Chinese group, 62.3% in German group), but Chinese patients seemed more likely to have lacune in regions of thalamus, brainstem or cerebellum than German patients (16.1% vs. 7.0% P < 0.01) which are close to posterior circulation area. With respect to microbleeds, a similar distribution could be detected: in the Chinese group, most of the microbleeds (44.6%) were located in deep regions, while microbleeds were more often located in lobar areas in the German group (62.4%). Furthermore, the comparison of the white matter changes in all regions demonstrated that the German group had a higher mean ARWMC score than the Chinese group in the subcortical region [8(6, 10) vs. 5(3, 8.5) P < 0.01], with no differences between the two samples in the basal ganglia and infratentorial regions.

## Discussion

A growing body of literature reports on ethnic disparities in stroke patients and CSVD between Western and Chinese populations^7,8,16^. Throughout the literature, Chinese patients are reported to have a younger age-onset of stroke and a high prevalence of CSVD. In this comparative study of CSVD between a Chinese and a German cohort, we found a younger age at onset of CSVD in Chinese patients and a different anatomical distribution of CSVD-associated lesions, including lacunes, white matter changes, and microbleeds. The observation that the German group presented with more lacunes (in deep and infratentorial regions) is not at odds with previous reports of a higher susceptibility to CSVD in Asian patients^5,7–9^, given that the current study compared CSVD patients and did not address the different etiologies in general stroke populations from the two countries. With respect to the different types of CSVD^1^, our study has focused on type 1 (arteriolosclerosis) and excluded other types of CSVD as far as possible in the final analysis. Further, patient groups between countries presented with significantly different cognitive profile which was attributed to differences in age.

To date, age and hypertension are the most commonly recognized risk factors of arteriosclerotic CSVD, while others such as diabetes, dyslipidemia, and smoking are still controversially discussed, possibly reflecting different underlying subtypes or pathogenesis^1,17^. The results of our comparison showed that there was no significant difference in the proportion of hypertension between the two groups, but a striking difference in age. According to statistics of the World Health Organization, in 2016, life expectancy in Germany was 81.0 years and 76.4 years in China^18^. The difference in life expectancy of about 4.6 years is less than the observed 10 years difference between the two groups in the current study, which points to an early onset of CSVD in Chinese patients. A similar age difference between other different ethnicities were observed in the Secondary Prevention of Small Subcortical Strokes (SPS3) Trial with 3,020 participants from 81 clinical sites in 8 countries which suggested a 6 year difference in the ages of CSVD onset with an average age of 58 years (SD = 9.5) for Black participants compared with 64 years for White (SD = 10.8) and Hispanics (SD = 10.7)^19^.

Beyond age, a marked difference between Chinese and German patients was the different spatial distribution of CSVD-associated findings. Although Chinese patients had fewer lacunes and microbleeds than German patients, Chinese patients presented with significantly more lacunes and microbleeds in deep and infratentorial regions. German patients had significantly more severe white matter changes in subcortical areas, but lacunes and microbleeds were centered preferentially in subcortical areas and lobes. The supratentorial and lobar distribution of lesions might explain poorer cognitive performance in neuropsychological tests. This result corresponds with the findings by Yakushiji and colleagues that the Eastern populations had higher odds of deep and/or infratentorial microbleeds than Western populations^12^. A different distribution of CSVD lesions between different ethnicities was also found in the SPS3 study^11^ which showed Blacks and Hispanics having more recent lacunar infarcts in the brainstem/cerebellum than non-Hispanic Whites (p < 0.001). Different risk factor profiles might be one reason for this finding. The Rotterdam Scan Study showed that total homocysteine levels were associated with silent brain infarcts and periventricular and subcortical white matter lesions^20^, while smoking and systolic hypertension was found to be associated with deep or infratentorial but not lobar microbleeds^21^. Hyperlipidemia was hypothesized as a relatively protective role in CSVD and was found to be associated with less severe WMC^22^. Obesity is recognized as an potential risk factor for CSVD and to be associated with incident lacunes^23^ and deep or infratentorial microbleeds^24^. In the current study, there were higher proportions of hyperlipidemia and smoking in Chinese patients but a higher rate of hyperhomocysteinemia and obesity in German patients which might correlate with the respective imaging changes at least to some extent.

Another important issue of consideration was the control of hypertension. Although the available data showed no differences in the proportion of hypertension between the two groups, literature reports suggest that there is a significant difference in hypertension control between the two countries. A population-based screening project that enrolled about 1.7 million adults in China from 2014 to 2017 showed that the age and sex standardized rates of hypertension awareness, treatment and control were 36·0% (35·8–36·2), 22·9% (22·7–23·0), and 5·7% (5·6–5·7)^25^, while the reported rates were 82%, 72%, and 51% in Germany 2008^26^. Meanwhile, the level of health care varies considerably between regions in China. The reported rates of hypertension control in Beijing were 21.5% in 2007^27^ and 31.6% in 2013–2014^28^, respectively, which were higher than China’s average, but still significantly lower than Germany’s average. As a result, the small cerebral vessels may suffer more and earlier impairment from hypertension. In the same way, differences in the control of hypertension could in part explain differences in neuroimaging. Chinese patients had a poor hypertension control, leading to a hypertension-related brain change, which brought more ischemic or hemorrhagic damage on basal ganglia, thalamus, brainstem, or cerebellum. In contrast, German patients can be expected to have received a good hypertension control according to literature, presenting with less hypertensive-related but possible age-related brain changes, i.e., more subcortical white matter damage and cognitive impairment. In addition, it may also explain why significantly more Chinese patients had to be excluded from the final analysis due to the diagnosis of large vessel stenosis, since uncontrolled hypertension is the core risk factor of atherosclerosis in large vessels. Unfortunately, the available data did not include information on the first diagnosis and control of hypertension in our patients which prompts further research for confirming this point.

In addition, genetic differences may play an important role regarding severity and distribution of CSVD in Chinese and German patients. The APOE ε4 phenotype was reported to relate to microbleeds in a strictly lobar location^21^, which may be found not only in patients of Alzheimer disease^29^, but also patients of arteriolosclerosis CSVD simultaneously. There are numerous genes which may have an impact on CSVD by impairing the blood brain barrier. The latter was confirmed to play a major role in the formation of several CSVD-related lesions (lacunar infarction, white matter hypertension)^3^. Rannikmae and colleagues observed an association between common variations in the COL4A2 gene and symptomatic small vessel disease, particularly deep intracerebral hemorrhage, in the ethnic group of individuals of European ancestry^30,31^. In addition, dysfunction of connexins (Cx), an important component of gap junctions, can affect the permeability of blood-brain barrier in various ways^32–35^, resulting in WMC or microbleeds different anatomical regions. Therefore, does ethnic differences in these genotypes contribute to imaging difference of CSVD between our two countries? There is no answer yet, which requires further exploration and demonstration in future genetic and autopsy studies.

The strength of the study is that we conducted a detailed comparison of the important neuroimaging features in a well characterized clinical sample of patients with CSVD from China and Germany. As an exploratory research, it reflects the ‘real life‘ data of CSVD in the two countries to a certain extent and provides a direction of future research. The study, however, was not without limitations. First, it was a hospital-based retrospective study. Inevitably, selection bias may affect the interpretation of the results. Furthermore, data acquisition did not follow a standard operating procedure, although we applied some unified definition to ensure consistency in the processes of analysis and comparison. Finally, lack of information on social factors like income and status of health insurance might impact on health and CSVD and confound our results. A prospective study is being planned for confirming our results and assumptions.

## Conclusion

In conclusion, clinical and imaging differences exist between Chinese and German CSVD patients in the observed population. Chinese patients tend to be younger and to have more lesions in deep and infratentorial structures, while German patients have more lesions in the subcortical white matter and are susceptible to cognition decline. These observed differences need to be confirmed and clarified by further prospective studies.
